# Rethinking malaria: Governance lessons from other disease programs

**DOI:** 10.1371/journal.pgph.0000966

**Published:** 2022-09-27

**Authors:** Kelechi Ohiri, Ifeyinwa Aniebo, Oluwafunmilayo Akinlade

**Affiliations:** 1 Health Strategy and Delivery Foundation, Abuja, Nigeria; 2 Harvard T. H Chan School of Public Health, Boston, Massachusetts, United States of America; 3 Department of Emergency Medicine, University of Virginia, Charlottesville, Virginia, United States of America; CSIR-Indian Institute of Chemcial Technology, INDIA

## Abstract

The global disruptions brought about by the COVID-19 pandemic as well as the stagnation of progress of global malaria elimination efforts have provided an opportunity to rethink several aspects of the global malaria program, including its governance at all levels, from the community to the nation and to the world. Approaching this issue requires an examination of the critical governance factors that affect malaria elimination as well as lessons that could be learned from the governance of other global health programs. The paper, therefore, first reviews malaria program governance challenges at the global, national, and sub-national levels. We then conducted a literature review of governance factors that affected four major global disease elimination programs; (1) the global smallpox eradication program; (2) polio eradication efforts (focus on Latin America); (3) the onchocerciasis eradication program; and (4) global COVID-19 pandemic control efforts. Based on this review, we identified eight comment governance themes that impact disease elimination programs. These include 1) International support and coordination; 2) Financing; 3) Data use for engagement and decision making, 4) Country ownership; 5) National program structure and management, 6) Community support/engagement; 7) Multisectoral engagement; and 8) Technology and innovation The paper then illustrates how these eight governance themes were factored in the four disease control programs, draws lessons and insights about the role of governance from these programs and outlines the implications for governance of malaria elimination efforts. The paper concludes by making recommendations for improving governance of malaria elimination programs and how the analyses of other global disease control programs can provide new ideas and inspiration for a more robust push towards malaria eradication.

## Introduction

In May 2015, the World Health Organization (WHO), through its Global Technical Strategy for Malaria 2016–2030 [1], provided a comprehensive framework to countries and development partners to scale up malaria responses and move towards elimination. This document set the target of reducing global malaria incidence and mortality rates by at least 90% by 2030. However, since 2015, the reduction in the global burden of malaria appears to have stagnated with only marginal annual reductions in the case burden. There has also been a slowing of the rate of decline of malaria case incidence (cases per 1000 population at risk) [2]. As progress stalled, the global community began recognizing the need to rethink the approach to malaria elimination, culminating in the WHO calling for an ‘aggressive new approach’ in the 10+1 countries with the highest malaria burden: the “High burden to high impact” country-led approach [3].

The Covid-19 pandemic also created another major obstacle to progress in reducing the global malaria burden, particularly in its diversion of human and financial resources essential for malaria services and interventions towards combating the pandemic. On the other hand, the pandemic also provides an opportunity to rethink the approach to malaria and learn from other programs that have successfully put in place, governance structures and processes in the control, eradication, or elimination of infectious diseases such as smallpox, polio, and onchocerciasis. Although these programs have different disease dynamics and interventions, there may be relevant and useful governance lessons that could be applied to the global malaria elimination program, since these programs have financial, political, administrative, and operational similarities. It is worth mentioning that whilst not all the disease programs this paper considers, successfully achieved global elimination/eradication (e.g., onchocerciasis, Covid-19), we believe there are lessons about the governance of these disease programs that the malaria elimination program would benefit from.

In this paper, we investigate governance issues that affect malaria elimination efforts by reviewing and identifying factors that can strengthen malaria program governance at the global, national and sub-national levels. The paper then presents the literature review and methods for learning key lessons from four other global disease control programs, and how eight common governance themes were identified. We then illustrated the impacts of eight themes in the four disease control programs and the implications for malaria elimination efforts. The paper concludes with a few recommendations about how these governance lessons can be used to strengthen malaria elimination globally.

### Malaria governance challenges

Governance in the health sector commonly refers to the use of formal and informal institutions, processes and rules by states, nonstate actors and intergovernmental organizations to manage challenges to improving health conditions [4]. The governance of malaria control and elimination typically involves many different players, and can result in competition for leadership, influence, and resources at the global, national and community levels. We briefly review some of the challenges at these three levels.

At the global level, the number and variety of global health problems on foreign policy agendas has increased and continues to expand [5]. This creates two main issues for global health governance. First, global health problems generate different levels of interest from countries and development partners. Countries tend to be more interested in problems that directly threaten their interests. This pattern can be seen in the level of attention given to direct, cross-border transmission of dangerous communicable diseases such as Ebola. On the other hand, diseases that do not involve such transmission (including noncommunicable diseases) are perceived to get less attention. Secondly, the need to prioritize resources and responses may create a zero-sum scenario, often resulting in disagreements about how priorities are established [6] and complaints about some disease programs getting a disproportionate share of attention and resources. It is not surprising that this paper is being written against a background of perceived diversion of attention and resources to combating the COVID-19 pandemic. Whilst malaria gets more attention on the global agenda than neglected tropical diseases, it does not get as much attention as HIV/AIDS or COVID-19. In fact, in West Africa, for example, donor support for malaria is seen to be waning [7].

At the country level, the governance of malaria can have a direct impact on elimination of the disease. In malaria endemic countries, the National Malaria Control/Elimination Program (NMCP/NMEP) is responsible for developing malaria policies and strategies and provides technical leadership for the Ministry of Health (MOH) with respect to malaria prevention and control [8]. Organizational structure (administrative location), the effectiveness of administrative processes (earmarking and financial control), and strong leadership (assertion of state ownership and resourcefulness of leaders in overcoming bottlenecks) appear to influence the performance of malaria programs [9]. In addition, the financing dynamics, particularly the balance (or lack thereof) between donor and domestic funding, may have an impact on the level of alignment of such funds with country’s needs and priorities. Recipient countries often have restricted autonomy over donor resource allocation (which could be quite significant and influential), hence limited power to make decisions on how best to use donor resources to implement malaria programs in their own countries [7].

At the community level, the main challenge is the level of ownership the community has over malaria programs. This affects how communities respond to the implementation of policies. When the views of the community, who are the primary participants of policy implementation, are not fully considered during policy development, they are less likely to take ownership of the interventions during implementation [10]. For example, communities may accept free Long-Lasting Insecticidal Nets (LLINs) but not use them correctly. Most successful public health programs have involved significant community engagement in co-creation and involvement in implementation.

## Methods

To better identify and analyze governance challenges in malaria elimination programs, this paper examined the governance experiences of other global disease control programs, and then sought to identify lessons for malaria governance, globally and within countries For the purpose of this paper, we examined four disease programs: 1) the global smallpox eradication program; (2) polio eradication efforts (with a focus on Latin America); (3) the onchocerciasis eradication program; and (4) global COVID-19 pandemic responses.

For the analysis, we first conducted a literature search on each disease control program and identified pivotal papers and publications that discuss governance. The search was conducted on Pubmed for the four diseases of interest. The original search utilized terms synonymous with all four diseases. Using smallpox for example, the search utilized Smallpox OR “small-pox” OR “variola major” “variola minor” OR “orthopoxvirus” OR “pox” (Table 1). This was repeated for polio, Onchocerciasis and Covid-19 (Table 2). The search also utilized the following terms: Eradication OR elimination OR success OR program OR lessons learnt OR case study OR disease control OR disease eradication OR disease elimination OR leadership OR programmatic OR governance, and interview OR “focus group” OR qualitative for all disease programs.

**Table 1:** Governance themes in disease control programs.

Governance Theme	Definition and Description
International support and coordination	Coordinated advocacy and action by institutions or countries towards a global goal, support by global champions
Financing	Significant resource mobilization and funding from countries/ institutions (both domestic and international)
Data use for engagement and decision making	The impartation, communication or exchange of information and insight, and its use in decision making
Country ownership	In-country leadership and action by national and subnational governments and other actors
National Program structure and management	The organization, leadership, and management of a country’s disease program at the national level
Community support/engagement	Support organized at the community level, involving community leaders, or other groups, e.g., religious, civil society organizations
Multisectoral engagement	Coordinated and collective action and involvement of other sectors (e.g., finance, private sector, environment) at all levels
Technology and innovation	Availability and diffusion of innovation (and research) including non-complex scientific/medical interventions

**Table 2:** A conceptual framework of factors associated with success of smallpox, polio, and onchocerciasis and COVID-19 disease programs.

Theme	Smallpox (Worldwide)	Polio (Latin America, The Caribbean, and Nigeria)	Onchocerciasis (Sub-Saharan Africa)	COVID-19 Pandemic (Global)	Implications for the global malaria eradication program
**International Support and Coordination**	• The Soviet Union played a key role in initiating the eradication program in 1958 through its deputy minister of health • Over 1.5 billion doses of vaccine produced in the Soviet Union for mass vaccination in 45 countries over 20 years of the smallpox eradication program. -Strong Global advocacy from US Govt -Engaging national leadership at World Health Assembly	• Strong international advocacy from US President Roosevelt. In 1938, created the March of Dimes • Latin America and Caribbean regional coordinated effort with leadership from PAHO Interagency Coordinating Committee (ICC) for LAC involving UNICEF, IDB, PAHO, USAID, Rotary International, and the Canadian Public Health Association, Polio in Nigeria had support from WHO, the US CDC, UNICEF, the Bill & Melinda Gates Foundation, and Rotary International.	• Strong international advocacy from World Bank president, McNamara after his 1972 visit to Africa • Development of the global Onchocerciasis Control Program (OCP) in 1974 • Development of the African Program for Onchocerciasis Control (APOC) in 1995. involved agencies (WB, FAO, UNDP, WHO), governments of 19 developing countries, 21 bilateral and multilateral donors, > 30 NGOs, Merck, > 100,000 rural African communities	• WHO and GAVI leadership and collaboration with regional disease control entities such as the Africa CDC, US CDC, GAVI, CEPI for the COVAX initiative • Access to COVID-19 Tools Accelerator (ACT-A) to promote equal access to tests, treatments and vaccines and support health systems globally *This global leadership was however, attenuated by the rise in nationalism, particularly in high-income countries.	Significant global advocacy for malaria, Would malaria benefit from a global political champion? A person? A country? Or is the multilateral financing enough? Would the focus on elimination or eradication resonate better politically than more nuanced approaches e.g., control?
**Financing**	• In 1966 the World Health Assembly (WHA) approved $2.4 million annually to support a 10-year smallpox eradication plan • Technical and financial support from the US Govt. $35 million over a 5-year period, approved by President Johnson as a special US contribution to a United Nations initiative called ‘International Cooperation Year’. • Cost between 1967–1979 was US$23 million. In total, donors provided US$98 million, while US$200 million came from the endemic countries.	• International financial commitment from PAHO, UNICEF, USAID, IDB, Rotary International, Canadian Public Health Association) contributed $110 million between 1987 and 1991. • Increased domestic resource mobilization. The first five years of the polio campaign cost $120 million: $74 million from national sources and $46 million from international donors	• Merck’s long-term donation of Mectizan • Financial support mobilized through World Bank and donor partners. • Commitments from 27 donors during the 28-year OCP project totaled $600 million. • APOC bears a total price tag of $180 million. Donor funding accounts for 75%, and African governments and NGOs the remaining 25% • Yearly cost of less than $1 per person protected	• Unprecedented resource mobilization globally (~$11.7 trillion) for pandemic control and impact mitigation through economic stimulus funding • The establishment of the COVAX facility • World Bank approved $12 billion for developing countries to finance the purchase and distribution of COVID-19 vaccines, tests, and treatments for their citizens • A US$2 billion UN coordinated global humanitarian response plan	Substantial existing financing through multilaterals and bilateral agencies What is being funded? Is there similar funding for other interventions? Innovation? Is there scope for more domestic financing in the face of economic constraints from the covid pandemic?
**Country ownership**	• Identified politically connected domestic champions • National healthcare workforce mobilization at all levels. • Embraced independent actions by countries to test approaches across different sociocultural and epidemiological contexts	• National government commitment. • In the first 5-year plans from 1987 to 1991, 80% of the $544.8 million budget for EPI was derived from national resources. This figure climbed to 90% in the second 5-year plan. • National vaccine day campaigns introduced and implemented • Establishment of "Operation Mop-Up" Nigeria created a presidential task force to lead the country’s response to the eradication of polio	• National Ministries of health coordination in APOC model (unlike vertical design of OCP). • APOC pioneered Community-Directed Treatment with Mectizan (ComDT), that was owned and driven by the countries	• Countries were in charge of their national response, although there was extensive exchange of knowledge across countries. • Response largely led by local health officials and organizations. External TA providers largely played supporting roles	Do the countries really own their strategies? Several countries have national malaria programs. have these evolved into government-funded, rapidly responsive programs?
**National Program structure and management**	• ‘Military-like’ approach to contact tracing • National Program leader assignment • Smallpox programs were integrated with health systems • Experimental learning facilitated identification of local solutions • Culture of problem-solving among staff with reputations for adaptability	• PAHO’s regional polio eradication campaign complemented routine immunization efforts • National ICC set up and replicated in-country • Utilized the polio elimination strategy to strengthen the national immunization programs through complete integration with the Expanded Program on Immunization (EPI) Nigeria’s Ministry of Health created Emergency Operations Centres (EOCs) to focus on the highest-priority interventions, improve coordination, and to manage the program’s overall performance.	• APOC was not implemented as a vertical program, but integrated within the Health System • The focus was at the community level, and it was the community and community leaders that drove most of the implementation.	• National programs headed by a high-level program leader, often reporting to the President, e.g., Anthony Fauci in the US; Supra-ministerial or ministerial level officials, Matt Hancock in the U.K.	Most NMEP programs are a housed in a unit within the ministry of health. For instance, in Nigeria, it is a program, that reports to the Director of Public Health, which reports to the permanent secretary which reports to the minister of health which reports to the president. Hence, not that much visibility or priority given their position. The NMEP program manager needs more visibility!
**Community support/ Community engagement**	• Large scale community mobilization through volunteers [11] • Community leaders’ support • Developed a network of agents who conducted active case detection activities • Combined mobilization efforts with other community initiatives (neonatal care, census taking, market days)	• Community-driven, house-to-house vaccination campaigns • Thousands of community healthcare workers were trained on tasks including surveillance, and cold chain management and mobilized across the country • Nigeria’s Polio program addressed the challenges of communication, social mobilization, and noncompliance. Supported traditional, religious, and opinion leaders, to overcome vaccination misinformation.	• Extensive community engagement and involvement in the implementation of Community-Directed Treatment with Mectizan (ComDT) • The communities selected the community-directed distributor, and the distribution efforts were adapted to the local culture and conditions. • Community volunteers received training and supervision from the national public health systems and from the program’s NGO partners.	• Engaging communities in the maintenance of pandemic prevention guidelines e.g., social distancing compliance, identification of cases • A significant part of the response has been top-down, given the nature of the pandemic • Successful behavior change modification interventions including hand sanitizing and mask wearing.	A lot of interventions are top-down, e.g., campaigns to distribute nets, testing, and treatment programs (albeit where access is limited). Do communities own this? Particularly vector control mechanisms to destroy breeding sites, environment. Do communities also understand the importance of malaria elimination? Do they see malaria as a problem?
**Use of data for engagement and decision making**	• **Case finding** intensified during the period of lowest seasonal incidence • Integrated reporting from health facilities and active surveillance. In India, surveillance augmented to focus on routine, repetitive active searches for cases. (90% of houses every two months.) • Shift from national mass vaccination to surveillance and focused vaccination in areas where smallpox was observed	• Establishment of **disease surveillance system** Established a surveillance network of about 22000 health facilities and labs Alignment of indicators to track including suspected cases and incentivizing their reporting ($100/case). Nigeria’s national EOC used a war room approach where the walls were covered with regularly updated wild poliovirus maps, data and analysis on polio cases, and polio immunity coverage in the country’s 11 high-risk states. The room used digital screens to depict up-to-date polio-performance indicators as well as videoconferences with state EOCs and external experts.	• Detailed geospatial mapping of 12,000 miles of rivers to provide up-to-date information • Detailed **epidemiological mapping** of the disease that aided surveillance. Operational research budget built into the program	• Regular simple presentation of data in a compelling and engaging manner • Use of platforms that increased access to real time sequencing data which contributed to rapid diagnostics development e.g., virological.org • Real time decision-making informed by data	Beyond MIS surveys (every 5 years) & World Malaria Report (modelled data). Could malaria data be used and presented in more engaging ways? Can we improve surveillance to include genomic and other high-quality data? Do we need systems/ platforms that provide real-time data? And do we need more frequent surveys e.g., yearly as opposed to every 5 years?
**Technology and Innovation**	• Invention and supply of the **bifurcated needle**: inexpensive, easy to use and required only a quarter of the vaccine dose normally required. • Rapid Training of vaccinators (took 15 min) and they could vaccinate 500/day. • **Freeze-dried vaccines**: Providing a fully potent, heat-stable vaccine which cost 1 or 2 cents a dose	• Development of the **inactivated Vaccine** • Development of the **Oral Polio Vaccine** and its effective deployment tin Chiapas, Mexico, served as a model for large scale immunization • Computerization of the surveillance system	• Helicopter-facilitated insecticide use • Discovery of Mectizan which relieves the agonizing itching of the infection and halts progression toward blindness.	• Rapid development and deployment of vaccines due to fast track of regulatory approval process e.g., European Medicines Agency (EMA). • Rapid rollout of tests • Use of technology to track and trace	• Perhaps if there was an improvement in the interventions? E.g., A vaccine? Single dose antimalarial drug? Newer approaches to treatment and prevention? Would elimination be more attainable? • Innovative financing mechanisms have not yet yielded the desired results.
**Multisectoral Collaboration**	• Engaged the private sector • UNICEF provided commercial-sized freeze-drying machines	• Establishment of the Interagency Coordinating Committee working across sectors	• Long-term public-private partnerships • The Private Sector role in the success. Merck (and the Carter Center) showed resilience in trying to engage public sector (WHO, USAID).	• Multisectoral national responses involving transport, security, education sectors • Private sector (/pharma) collaborating with regulatory agencies e.g., European Medicines Agency (EMA).	• Private sector is engaged, however, not always for purely altruistic reasons. • How effectively can we bring the private sector to better partner with the government beyond CSR? • Which sectors should be brought to the table? Environment? Education? Water and Sanitation? Mining? Agriculture?
Selected References	CASE 1: Eradicating smallpox. https://www.cgdev.org/page/case-1-eradicating-smallpox Henderson DA, Klepac P. 2013 Lessons from the eradication of smallpox: an interview with D. A. Henderson. Phil Trans R Soc B 368: 20130113. http://dx.doi.org/10.1098/rstb.2013.0113 Shchelkunova GA, Shchelkunov SN. 40 Years without Smallpox. Acta Naturae. 2017 Oct-Dec;9(4):4–12. PMID: 29340212; PMCID: PMC5762823.	CASE 5: Eliminating polio in Latin America and the Caribbean. (n.d.). https://www.cgdev.org/page/case-5-eliminating-polio-latin-america-and-caribbean Henderson DA, Klepac P. 2013 Lessons from the eradication of smallpox: an interview with D. A. Henderson. Phil Trans R Soc B 368: 20130113. http://dx.doi.org/10.1098/rstb.2013.0113 https://www.mckinsey.com/industries/healthcare-systems-and-services/our-insights/eradicating-polio-in-nigeria#	CASE 7: Controlling onchocerciasis in sub-Saharan Africa. (n.d.). Retrieved from https://www.cgdev.org/page/case-7-controlling-onchocerciasis-sub-saharan-africa	https://www.who.int/initiatives/act-accelerator www.gavi.org https://virological.org/ www.cdc.gov https://www.businesstoday.in/current/world/global-cost-of-coronavirus-this-is-how-much-covid19-pandemic-has-cost-the-world-economy/story/425100.html	

The search terms could appear anywhere in the full text of the paper (including title, abstract, text, keywords), and limits were applied by date (1980-August 2021 for smallpox, 1999- August 2021 for Polio, 1958- August 2021 for onchocerciasis, and January 2020- August 2021 for Covid-19). Only papers reporting research on human subjects in English language were included. The search was carried out by two independent reviewers.

**Fig 1:**
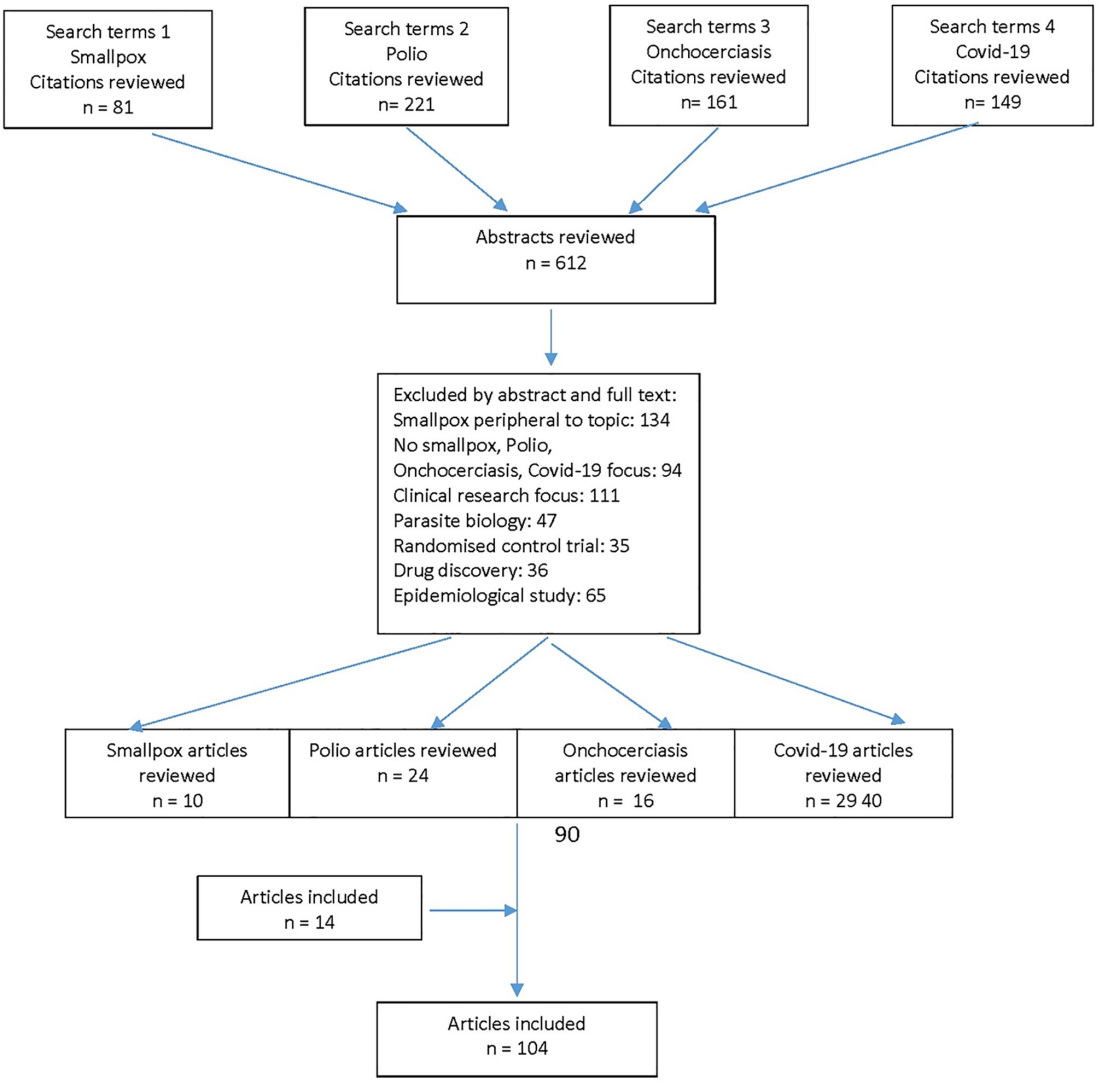
Search methodology.

Coupling the search terms for all four diseases (Fig 1) identified 612 articles. After review of titles and abstracts, 522 articles were considered potentially relevant and retrieved for full text review. 90 articles were considered relevant to the objectives of this review. 14 additional relevant articles were identified either from a simple desk search or reference lists of retrieved articles. These searches collectively provide information that assist in the understanding of governance themes for all four diseases, and highlights lessons learnt from these disease programs which the malaria elimination program could learn from.

We then examined the papers to identify common governance themes throughout the papers for the four disease program areas of interest. Eight governance themes that were common across the disease programs. (Table 1) presents the eight themes and illustrates each theme with examples from the four disease control programs.

Our analysis of these four disease programs recognizes that they differ in many ways. For instance, they involve different pathogens (some are viruses, whilst some bacteria), they affect different geographic regions/populations, and some are yet to be fully eradicated (such as onchocerciasis and COVID-19) Nonetheless, we believe that there are still governance lessons to draw from these programs that could be applicable to the malaria elimination efforts. The next section presents key lessons for each of the eight governance themes, with illustrations from the four disease control programs and proposed implications for malaria efforts.

## Results: Key lessons for the eight governance themes

### 1. International support and coordination

One of the main features of these programs was the high level of international collaboration, advocacy and support that galvanized the world to prioritize and tackle these issues. International coordination was considered important to avoid “ping-pong smallpox” [12] in which infections would be continually reintroduced from country to country. The smallpox program survived and was successful in part because it had international support and strong backing from the major powers of the era, the United States, and the Soviet Union [13]. There was no common pattern to the origin of such international support, beyond the presence of an influential global leader who made the programs part of their legacy. In the case of smallpox, the eradication effort coincided with the reemergence of the Soviet Union on the global scene and the opportunity to exercise some soft power, through the then Deputy Minister of Health. It helped that the United States was also fully engaged, and its presidents emerged as champions for these causes. (For example, President Roosevelt created the March of Dimes to support polio eradication and President Johnson sought to lead efforts by the UN and provided support to the smallpox eradication.) In other instances, it was technocrats, such as World Bank President Robert McNamara, who supported the onchocerciasis program after a visit to Burkina Faso in 1972.

With regards to COVID-19, we have seen both some degree of global solidarity–through the establishment of the COVAX facility–but also a lack of global cooperation through increasing vaccine nationalism. This suggests three lessons about international support and collaboration: Firstly, the global champion or influencer plays a critical role by promoting and pushing international cooperation as a legacy. Secondly, global efforts need to be anchored within a multilateral organization (such as WHO or the World Bank) or regional organizations such as the Africa CDC [14] to convene the best minds and to organize operations to achieve this goal in a short to medium term. Thirdly, global collaboration is critical for success (over public health nationalism).

#### Implications for malaria

The global malaria program needs to identify a global champion (perhaps a world leader or head of an influential global organization) who can accelerate and promote elimination as a global priority. Questions that the global malaria community need to reflect on include: Would malaria benefit from a global political champion? Would the focus on elimination or eradication resonate better politically than more nuanced approaches e.g., control? These should be considered in the next global strategy.

### 2. Financing

Closely linked to global advocacy is international and domestic resource mobilization to support the global efforts at disease control and elimination. There was international financial support for smallpox, polio, and onchocerciasis from a combination of players in global health, ranging from multilateral institutions to the private sector. For example, in the case of smallpox, in addition to the countries committing resources to the eradication effort, there was significant resource mobilization by the international community. The WHO provided a dedicated smallpox funding in 1967 which incentivized countries to scale up their national programs [15]. The World Health Assembly (WHA) committed to a minimum annual spend over 10 years, and the US committed 5-year financing. However, domestic resources from countries with smallpox also played a large role, as more than two-thirds of the financing between 1967 and 1978 came from endemic countries. A similar situation occurred in Latin America’s polio eradication program, where endemic countries contributed $74 million of the $120 million spent in the first five years of the program. The program received financial and logistical support from partners such as the WHO, UNICEF, CDC, the Task Force for Global Health, Rotary International, and Gavi [16], which facilitated advocacy and social mobilization. With the COVID-19 pandemic, the world has witnessed unprecedented resource mobilization for the health response, as well as for financing to cushion the impact on the economy (micro and macro). Most of this is at the national level, but internationally, a lot of financing has also been mobilized [16].

#### Implications for malaria

Key questions for malaria (for both donors as well as national governments of endemic countries) are: whether current funding is enough, given the global burden; whether current funding levels can be sustained, given other demands; and if current funding is being effectively utilized? These questions require coherent and persuasive responses from the global malaria community. For example, malaria programs today frequently experience challenges with expenditure, including delays. Grants from the Global Fund to Fight AIDS, Tuberculosis, and Malaria are not spent on schedule in many countries due to various reasons, such as weak data systems, delays in procurement, and lack of human resources. The smallpox eradication program created a flexible fund to address implementation bottlenecks in endemic countries as they arose. This method could be applied to malaria elimination programs, provided there is sufficient transparency and accountability to ensure that funds are spent for their intended function. Investment in local manufacturing as a means of reducing dependence on donor-funded commodities (such as bed nets) may also need to be considered.

Efforts must also be made to reduce the cost of eradicating malaria and make it more affordable. One reason for the pivot away from earlier efforts (in the 1960s) at malaria eradication to smallpox was the cost of the program per person. According to a reported interview with D.A. Henderson, the malaria program accounted for over 20% of all funds available to WHO in the 1960s [17]. This was perceived as unsustainable as it resulted in less funding being available for other programs, coupled with the realization that eradication would be more costly and take longer than planned. The onchocerciasis eradication program on the other hand, cost $1 per person protected, and the smallpox vaccine cost 1–2 cents per dose.

### 3. Country ownership

Independent actions by countries to test many approaches simultaneously across different sociocultural and epidemiological contexts was an important success factor for other disease control programs. For example, the global smallpox eradication effort was built on leadership and support from WHO, but in practice was a collection of individual national programs attempting to solve their own problems through their own systems and in their own ways [18]. Experimental learning rather than formalized programming was encouraged, and this facilitated the identification of local solutions. This is somewhat different from the way donor financing for several malaria programs currently operate. Smallpox’s profile within the WHO was maintained, and countries were encouraged to contribute funding and resources. The annual meeting of the WHO assembly was an important opportunity to keep eradication on the minds of health ministers [19] and surveillance reports with summaries of progress and problems was used to maintain the public profile of the disease. The smallpox eradication effort was successful also because it was a collection of individual national programs, each contextualizing solutions to their own [20], rather than a top-down, centrally managed approach [21].

#### Implications for malaria

Malaria endemic countries need to be encouraged to test various context-appropriate strategies while encouraging adoption of proven best practices. Although current malaria guidance embraces the belief that adapting and tailoring interventions to the local context is important for elimination success [22], the reality often does not match the rhetoric. Resources are deployed in ways that result in the recipient countries not having full autonomy over malaria policy and resource allocation; therefore, they cannot make decisions on how best to implement malaria prevention, diagnosis, and treatment in their own countries [7]. The existence of multiple players in malaria at the global level also contributes to competition for leadership, influence, and resources at the national level [23]. Country ownership is important, for example, Zambia takes ownership, makes decisions, and provides evidence to the global entity to change policy. One of the reasons for this is the maturity and strength of Zambia’s NMEP, which enables its staff to make decisions. This is emphasized in the country’s creation of a technical working group formed to avoid clashes in governance that may occur between partners at the global and national level. In situations where the technical working group’s decisions are challenged or pushed back by partners at the global level, the malaria manager makes the final decisions.

### 4. National program structure and management

Successful disease programs have strong management, integration in the national health system, and buy-in by top political decision makers. These programs also integrated their control structures within the country’s health systems in ways that strengthened national systems. Successful execution of the smallpox program, for example, was said to consist of 10% technical skill and 90% organization and leadership skills [24], with its approach to certain interventions such as contact tracing often described as ‘military-like’. Smallpox eradication had problem-solving staff with reputations for adaptability, imagination, and hard work; they served as catalysts, rather than controllers, and strong managers and operations officers were hired to ensure execution. Some factors were important for elimination of smallpox. First, smallpox programs were integrated with basic health systems, which allowed case management and surveillance to occur on a routine basis [13]. Second, smallpox programs had staff who were creative problem-solvers [21, 25], and who could figure out how to overcome any obstacle that arose, thereby adapting solutions challenges faced [15]. Third, the smallpox program highlighted the importance of strong management in all aspects of the program [26]. The polio eradication initiative was also used to strengthen national immunization programs in Latin America. Some successful disease control programs (including COVID-19 responses) have leveraged proximity to top political leaders effectively, for instance in Nigeria, there was a Presidential task force on Polio. Most National Malaria programs are currently housed within departments in the MoH, which constrains their ability to galvanize political support and multisectoral action.

#### Implications for malaria

The management and leadership skills of National Program Managers need to be strengthened for successful program implementation. NMCP/NMEP managers need to have the right level of skills and visibility to be effective, including engaging with communities, problem solving, and creating context-appropriate solutions to problems that may arise. Managers usually don’t have enough training on leadership/management, and most are put in new positions based on their technical expertise and experience.

### 5. Community engagement

Community engagement and participation were critical for these global disease programs. Top-down approaches alone, have limited effectiveness. **For the polio eradication program, health worker mobilization played an impactful role in providing human resources that went house-to-house in communities with existing polio cases or had low coverage [27]**. Community participation with the smallpox program was considered to be strong [6]. Gaining the support of the community leaders was an important step towards community acceptance. Polio and smallpox efforts in Nigeria, for example, were successful because community/religious leaders trusted by communities were enlisted and engaged as part of the program [28]. For the APOC program, extensive community engagement and involvement in the implementation of Community-Directed Treatment with Mectizan (ComDT) contributed to its success [29]. Engaging the community should not be limited to a specific disease program but involve building capabilities to provide broader health services. In the smallpox eradication program, there were combined mobilization efforts with other community initiatives (e.g., neonatal care). For the polio eradication program, the training the community volunteers received included training on disease surveillance and cold chain management.

#### Implications for malaria

Malaria programs should engage communities and community leaders in ways that complement existing top-down approaches such as campaigns to distribute nets. Communities need to understand and own the issues and the interventions. For instance, do communities understand and own vector control mechanisms to destroy breeding sites in their environment? Do communities also understand and own the goal of malaria elimination? There should also be continuous communication and collaboration with communities as real partners in the conceptualization, design, and implementation of malaria elimination programs.

### 6. Data use for engagement and decision-making

The availability of real-time, high-quality data for surveillance and monitoring was a critical success factor for the disease eradication programs. In the polio eradication program, over 20,000 facilities were included in the surveillance network and an emphasis was placed on surveillance to track outbreaks, facilitated by the surveillance system’s computerization [27]. In the APOC program, epidemiological mapping techniques were used to map 12,000 miles of rivers for the program [29]. The COVID-19 response also effectively leveraged technology and data. Real time epidemiological data was used to efficiently align program strategy and deploy interventions in many countries. The smallpox program used surveillance data to seek out cases and then vaccination efforts were concentrated to those in their proximity and their contacts [30]. The surveillance strategy helped focus vaccination on the places where it was most likely needed, rather than laboring to achieve implausibly perfect coverage everywhere. This contributed to eradication’s ultimate success [31, 32]. Data were also used effectively to engage the population and various stakeholder groups in a simple and compelling manner. For instance, the COVID response programs in different countries used simple dashboards that were updated daily, to inform and engage citizens on the evolution of the pandemic, the progress made, and risks, for instance, epidemiological assessments informed the control measures that were implemented and the epidemic in Wuhan was under control within 100 days [33].

#### Implications for malaria

Malaria programs need to provide more frequent high quality malaria data at the national, state and community levels, and to use data to engage stakeholders and target interventions. Malaria programs should focus more on impacts and outcomes, including more frequent measurements of prevalence and incidence (which are directly linked to eradication) and perhaps less on outputs and activities conducted. Such data can be used in better engagement with stakeholders and communities on the status of eradication efforts. Unfortunately, the malaria indicator survey (MIS) is carried out every five years, which is not frequent enough. Performance indicators from programs could also be better targeted, for instance not just on number of nets delivered, but on whether nets are delivered to those most at risk, or if the nets achieve the desired outcome of reductions in malaria prevalence/incidence in the target communities. Questions the global malaria community may need to reflect on include: Can malaria data be used and presented in more engaging ways? To what extent should malaria programs rely heavily on modelling estimates to make decisions? Can we improve surveillance to include genomic data and other high-quality data in real time or with greater frequency?

### 7. Multisectoral collaboration

Lessons learned from diseases like Covid-19 show multisectoral collaboration for instance between governments and the biopharmaceutical industry via fast tracked regulatory approval process that led to the rapid development and deployment of vaccines was critical to control the spread of infectious diseases as well as mitigate its impact on populations [33, 34]. The relevant sectors span healthcare, education, research & development, tourism, and others. Most national COVID-19 responses have been multisectoral in nature, involving coordination of several public sector line ministries as well as the private sector. Pharmaceutical companies have been in public-private collaboration with governments, regulatory agencies, research institutions and international organizations. Other successful programs also involved the private sector, for example, Merck was highly involved in both the APOC and onchocerciasis control programs (OCP) [29].

#### Implications for malaria

Successful malaria elimination programs also involved multisectoral collaboration in their malaria strategic plan. For example, Zambia works with multiple sectors for malaria elimination, such as the mining industry and civil society. In fact, Zambia created a multisectoral ‘end malaria council’ to deepen its multisectoral approach. This involved representatives from various sectors including the private sector and development partners. Other countries might find this multisectoral approach coordination and governance approach useful.

### 8. Technology and innovation

Innovation played a crucial role in the success of some global programs by transforming the options available for interventions and thereby accelerating disease eradication. In the smallpox program, two innovations were pivotal. One was an inexpensive bifurcated needle that was easy to use and required only a quarter of the vaccine dose normally required [29]. The second innovation was freeze-dried vaccines that provided fully potent heat-stable vaccines that could be stored for months [35]. The innovation of the discovery of the drug Mectizan was at the heart of the APOC program [29]. Successfully eradicating Polio in Latin America and the Caribbean was a global, collaborative feat. Some critical factors for success were international support, the development of the inactivated polio vaccine (IPV), and massive community health worker mobilization [16, 27]. In addition, the Smallpox programs relied upon having a stable, reliable, effective vaccine [36]. In the fight against COVID-19, the rapid, unprecedented development and deployment of vaccines has been the game-changer in the global fight against the pandemic.

#### Implications for malaria

Innovations in the available interventions may accelerate attainment of malaria eradication goals. For example, an effective vaccine could be a game-changer–a new malaria vaccine showed about 77 percent efficacy in a small clinical trial among children in Burkina Faso, shows some promise in this regard [37]. A single-dose antimalarial drug could also radically improve treatment options.

## Conclusion

There is no ‘ideal program’ that can be directly compared to the malaria elimination program, as each has contextual issues, success factors and challenges. However, some governance lessons from other programs could provide new ideas and inspiration for a more robust push towards malaria elimination.

Some of these learnings are as follows: Firstly, the role of the sponsor or global champion is important; although the malaria program has many champions, it would benefit from having a global leader who makes this his/her priority and legacy. Secondly, national programs (and the international institutions that support them) must embrace flexibility and efficiency in execution and must be adaptive in their approach at all levels including the way stakeholders such as political leaders, other sectors, and the community are engaged. Thirdly, successful programs highlight extensive community engagement and involvement in the implementation of interventions, including behavioral change modifications. Fourthly, there is an opportunity to rethink the type of data being collected, its frequency, and its use in engaging stakeholders. Lastly, whereas other programs have clear mandates to eradicate the diseases, resulting in a focused, almost binary approach to measuring success–eradicated or not–success for the malaria program seems to be more complex, with eradication, elimination, and control as parallel, simultaneous goals. This may be pragmatic at a national level but may not have the same political resonance as a clear, single focus on global eradication.

## Supporting information

S1 Text "Rethinking malaria in the context of COVID–19," a global engagement organized by Harvard University. (DOCX)

S2 Text Synopsis case studies from other programs. (DOCX)

S1 Table Expert panel table comprising list of stakeholders and key experts. (DOCX)

S1 Checklist (DOCX)
