# Supplementary material for: Rethinking malaria: Governance lessons from other disease programs
Source: PLOS Glob Public Health. 2022 Sep 27;2(9):e0000966. doi: 10.1371/journal.pgph.0000966 (PMC10021884; doi:10.1371/journal.pgph.0000966)
Supplement: S2 Text — (DOCX) [file pgph.0000966.s002.docx]

**S2 Text: Synopsis of Case Studies from other Disease Programs: Global Smallpox Eradication, Polio Eradication in Latin America and the Caribbean, Onchocerciasis Control in Sub-Saharan Africa, and the Ongoing COVID-19 Response Globally.**

Case Studies

1. Smallpox Eradication

The Intensified Smallpox Eradication Programme of 1966–1977 was a global effort to conduct mass vaccination in combination with surveillance to detect cases and control outbreaks [21] and as a result, Smallpox is the only infectious disease of humans to have been eradicated globally. The smallpox program survived and was successful in part because it had international support and strong backing from the major powers of the era, the United States and the Soviet Union [22]. Smallpox’s profile within the WHO was maintained, and countries were encouraged to contribute funding and resources. The annual meeting of the WHO assembly was an important opportunity to keep eradication on the minds of health ministers [23] and surveillance reports with summaries of progress and problems was used to maintain the public profile of the disease.

International coordination was considered important to avoid “ping-pong smallpox” [24] in which infections would be continually reintroduced from country to country. The WHO provided a dedicated smallpox funding in 1967 which incentivized countries to scale up their national programs [25]. The smallpox eradication effort was successful also because it was a collection of individual national programs, each contextualizing solutions to their own [26], rather than a top-down, centrally managed approach [27]. Smallpox programs relied upon having a stable, reliable, effective vaccine [28] and the WHO provided an analogous quality control and assurance function. The WHO also provided technical and operational support to national programs and encouraged research and innovation through the development of a heat-resistant vaccine. Which was the single most impact factor in global success. The Smallpox program highlighted the importance of problem-oriented research which enabled resolution of unforeseen challenges [29].

Some factors were important for elimination of smallpox. First, smallpox programs were integrated with basic health systems, which allowed case management and surveillance to occur on a routine basis [22]. Second, smallpox programs had staff who were creative problem-solvers [27], and who could figure out how to overcome any obstacle that arose, thereby adapting solutions challenges faced [25]. Third, the smallpox program highlighted the importance of strong management in all aspects of the program [30].

1. Polio Eradication in Latin America and the Caribbean Success Factors

Successfully eradicating Polio in Latin America and the Caribbean was a global, collaborative feat. Some critical factors for success were international support, the development of the inactivated polio vaccine (IPV), and community health worker mobilization [31, 32]. The program received financial and logistical support from partners such as the WHO, UNICEF, CDC, the Task Force for Global Health, Rotary International, and Gavi [32], which facilitated advocacy and social mobilization. International collaboration spearheaded by the Pan American Health Organization (PAHO)’s regional polio eradication campaign complemented routine immunization efforts by utilizing the polio elimination strategy to strengthen the national immunization programs through complete integration with the Expanded Program on Immunization (EPI) [31,32]. PAHO also developed the PAHO IPV Introduction Practical Guide and expanded on resources developed by the Immunization Management Group (IMG) of the GPEI to support countries in introducing the IPV vaccine [32]. Health worker mobilization played an impactful role in providing human resources that went house-to-house in communities with existing polio cases or had low coverage [31]. Finally, an emphasis was placed on surveillance to track outbreaks, facilitated by the surveillance system's computerization [31].

1. Onchocerciasis Control in Sub-Saharan Africa Success Factors

In 1995, African Programme for Onchocerciasis Control (APOC) was established to advance the progress of the Onchocerciasis Control Program (OCP) started in 1974, which addressed disease vector control through environmentally safe Aerial insecticide use weekly [20]. APOC, founded to control Onchocerciasis in 19 central, east, and southern African countries, successfully partnered with local communities and international organizations to achieve a broad, sustained impact. Some critical facilitators of this process were the regional coordination necessary to achieve vector control, financial support mobilized through the World Bank and the River Blindness Foundation, and Community-Directed Treatment with Mectizan (ComDT) approach implementation [20]. Through a comprehensive partnership, APOC and OCP successfully distributed Ivermectin (Mectizan) donated by Merck & Co., Inc., which prevents and cures the disease with a single dose to over 45 million people in Sub-Saharan Africa. The ease of this intervention coupled with Merck's long-term donation and efficient vector control helped ensure the program's sustainability. Operational research-based decision making [20] to explore developing problems ensured context-specific solutions.

1. Ongoing global COVID-19 response

In late December 2019, it was announced that there were some pneumonia cases of unknown etiology (PUE) in Wuhan City, Hubei Province, China, and the causative agent was quickly revealed to be a novel coronavirus (later named SARS-CoV-2). The incubation time was quickly assessed to be 3-7 days by the China CDC team. The team also assessed the major transmission routes of the virus, being respiratory and close contacts through droplets. Based on epidemiological assessment, control measures were implemented and the epidemic in Wuhan was under control within 100 days [33]

Countries such as New Zealand, Germany, and Taiwan, had swift public health measures put in place to control the outbreak and maintain low numbers. New Zealand implemented the most stringent lockdown of any country when they had only 102 COVID-19 cases and no deaths. The politicians trusted the scientists and the prime minister used powerful empathetic communication to engage the public with the response. After 7 weeks, New Zealand emerged virus-free. The country protected its most vulnerable populations and achieved the lowest COVID-19 mortality rate in the OECD. Other measures included early decisive lockdown measures; implementation of surveillance systems; use of masks; targeted testing strategies; and the use of information technology [34].

Africa showed unified leadership. The first case of the coronavirus was confirmed on February 14, 2020, in Egypt. One week later, on February 22, H.E. Moussa Faki Mahamat, the chairperson of the African Union Commission, convened an emergency meeting of ministers of health with all 55 member states in attendance. The meeting led to creation of the Africa Joint Continental Strategy for COVID-19 Outbreak. The coordination by the AU and member states led to the advancement of contact-tracing, surveillance, case management, and scaling up of testing across the continent. The alignment in vision helped build the capacity of member states to respond to COVID-19 through the Africa CDC. [35]

The factors that have been responsible for COVID-19 success include: (1) strong political will; (2) active case finding and quarantine of close contacts; (3) science-based guidelines for prevention, control, and treatment; (4) public compliance with mask-wearing, social distancing, and hand-washing; (5) public understanding and involvement (6) restricting public gathering and movement; (7) nucleic acid testing for a wider population once an outbreak was noticed; (8) Data sharing and accessibility; and (9) Rapid development and deployment of vaccines via fast tracked regulatory approval process [33, 34].
