# Supplementary material for: Rethinking malaria: Governance lessons from other disease programs
Source: PLOS Glob Public Health. 2022 Sep 27;2(9):e0000966. doi: 10.1371/journal.pgph.0000966 (PMC10021884; doi:10.1371/journal.pgph.0000966)
Supplement: S1 Table — (DOCX) [file pgph.0000966.s003.docx]

**S1 Table: Expert Panel Table**

**Key Stakeholders and Global Malaria Experts**

| **Expert Panel** | **Job title** |
| --- | --- |
| Professor Evelyn Korkor Ansah | Professor of Clinical Epidemiology  Director, Centre for Malaria Research  University of Health and Allied Sciences, Ghana |
| Dr Jesse B. Bump | Executive Director  Takemi Program in International Health  Lecturer on Global Health Policy  Department of Global Health and Population Harvard T.H. Chan School of Public Health |
| Professor Núria Casamitjana | Director of Training and Education at ISGlobal and Professor at the University of Barcelona. |
| Professor Marcia Castro | Andelot Professor of Demography and Chair Department of Global Health and Population Harvard T.H. Chan School of Public Health |
| Dr Nii Ayite Coleman | Consultant in health systems policy, financing, and governance. |
| Dr Anya Guyer | Global Public Health Consultant |
| Dr Margaret Gyapong | Director of the Institute of Health Research (IHR) and Coordinator of the Centre for Health Policy and Implementation Research (CHPIR) at the University of Health and Allied Sciences (UHAS), Ghana. |
| Dr. Maurice Itoe | Postdoctoral Research Associate. Harvard T.H. Chan School of Public Health |
| Dr Specioza Naigaga Wandira Kazibwe | Senior Presidential Advisor Population & Health · Ministry of Health -Uganda  Member African Union Panel of the Wise. |
| Professor Charles Mbogo | Public health entomologist |
| Professor Corrina Moucheraud | Associate Professor Department of Health Policy and Management University of California, Los Angeles. Associate Director, UCLA Center for Health Policy Research |
| Dr Halima Mwenesi | Malaria Expert and Global Health Consultant |
| Dr Kelechi Ohiri | Chief Executive Officer  Health Strategy and Delivery Foundation, Nigeria  Visiting Scientist, Department of Immunology & Infectious Diseases  Harvard T. H. Chan School of Public Health |
| Professor Friday Okonofua | Professor Friday Okonofua  Department of Obstetrics and Gynecology  University of Benin, Nigeria |
| Dr Fredros Okumu | Director of science at the Ifakara Health Institute in Tanzania. |
| Dr Jimmy Opigo | Head of National Malaria Control Programme. Ministry of Health, Kampala, Uganda. |
| Dr Ravindra Rannan-Eliya | Dr. Ravindra P. Rannan-Eliya is Executive Director & Fellow of the Institute of Health (IHP). |
| Professor Michael R. Reich | Taro Takemi Research Professor of International Health Policy at the Harvard T.H. Chan School of Public Health in Boston, Massachusetts |
| Professor Marcel Tanner | Former Director of the Swiss Tropical & Public Health formerly Swiss Tropical Institute (STI) and Professor, Chair of Epidemiology and Parasitology of the University of Basel. |
